# Local Stressors, Resilience, and Shifting Baselines on Coral Reefs

**DOI:** 10.1371/journal.pone.0166319

**Published:** 2016-11-30

**Authors:** Matthew McLean, Javier Cuetos-Bueno, Osamu Nedlic, Marston Luckymiss, Peter Houk

**Affiliations:** 1 Marine Laboratory, University of Guam, Mangilao, Guam; 2 Kosrae Conservation and Safety Office, Tofol, Kosrae State, Federated States of Micronesia; Department of Agriculture and Water Resources, AUSTRALIA

## Abstract

Understanding how and why coral reefs have changed over the last twenty to thirty years is crucial for sustaining coral-reef resilience. We used a historical baseline from Kosrae, a typical small island in Micronesia, to examine changes in fish and coral assemblages since 1986. We found that natural gradients in the spatial distribution of fish and coral assemblages have become amplified, as island geography is now a stronger determinant of species abundance patterns, and habitat forming *Acropora* corals and large-bodied fishes that were once common on the leeward side of the island have become scarce. A proxy for fishing access best predicted the relative change in fish assemblage condition over time, and in turn, declining fish condition was the only factor correlated with declining coral condition, suggesting overfishing may have reduced ecosystem resilience. Additionally, a proxy for watershed pollution predicted modern coral assemblage condition, suggesting pollution is also reducing resilience in densely populated areas. Altogether, it appears that unsustainable fishing reduced ecosystem resilience, as fish composition has shifted to smaller species in lower trophic levels, driven by losses of large predators and herbivores. While prior literature and anecdotal reports indicate that major disturbance events have been rare in Kosrae, small localized disturbances coupled with reduced resilience may have slowly degraded reef condition through time. Improving coral-reef resilience in the face of climate change will therefore require improved understanding and management of growing artisanal fishing pressure and watershed pollution.

## Introduction

“Shifting baselines” refer to successive generations of people having different baseline perspectives of the same ecosystem, because the ecosystem has changed drastically through time [1–3]. Shifting baselines are ubiquitous for coral reefs because widespread degradation has occurred over the last thirty to forty years [4–6]. For instance, over three decades, average coral cover has declined by roughly 80% in the Caribbean and 50% on the Great Barrier Reef [7,8]. Reef declines have various causes, but are generally considered to result from a combination of local stressors and disturbances [9,10]. Local stressors such as overfishing and pollution degrade ecological function and reduce ecosystem resilience, while disturbances such as coral bleaching or cyclones damage living coral cover [11–15]. Reefs suffering from local stressors are less likely to recover from disturbances and may become degraded through time, resulting in shifting baselines [9,10,16,17]. Because multiple stressors can affect reefs simultaneously and synergistically, understanding which local stressors are most responsible for ecological degradation is crucial for management.

While previous studies have linked large-scale coral mortality to major disturbances, or small-scale spatial patterns to local stressors [7,8,18–20], fewer have linked temporal changes in fish and coral community structure with local stressor gradients, especially over multiple decades [21, 22]. Studies documenting shifting baselines in fish and coral assemblages highlight ecological patterns, but often fail to evaluate long-term drivers, because historical data are scarce and difficult to integrate [23]. Alternatively, studies examining the influence of local stressors on reef health tend to evaluate spatial patterns within a single timeframe, but rarely track how stressors affect long-term reef changes [24,25]. And while more studies have begun to evaluate temporal changes in fish and coral assemblages along local stressor gradients, most do not span multiple decades [16,26]. As a result, scientists generally agree that reefs are in decline, but disagree on the magnitude of individual drivers of decline and how to prevent them [11,27,28]. Three prominent drivers of reef decline can be synthesized from existing literature, which many not be mutually exclusive: 1) increased disturbance frequencies from climate change [29–31], 2) pollution [32–34], and 3) fishing [15,35,36]. Identifying the relative magnitude of these potentially synergistic drivers of decline is important because previous studies have concluded that our ability to mitigate global change is negligible compared to our ability to manage local stressors [37,38]. Furthermore, decoupling the relative influence of local stressors and disturbances is difficult, and studies that examine the influence of local stressors while accounting for disturbances are needed.

This study used a historical baseline to compare the spatial structure of fish and coral assemblages between two separate surveys, one in 1986 and one in 2015, in Kosrae, Federated States of Micronesia. We first characterized fish and coral assemblages for both time frames with respect to wave energy, watershed size, and proxies for local stressors—fishing access and pollution. We then developed latent variables to (i) assess overall fish and coral assemblage condition, (ii) rank sites by their fish and coral assemblage condition scores, and (iii) assess the influence of local stressors on site rankings. Last, we evaluated differences in site rankings between timeframes to assess spatial changes through time, and test whether natural or human factors were related to these changes. This approach examined whether local stressors could predict changes in fish and coral assemblages, while controlling for different survey techniques that often exist with historical datasets. Ultimately, the present study reveals how coral-reef resilience may have shifted through time in Kosrae, and recommends where to focus future science and management efforts.

## Materials and Methods

### Ethics statement

Field work and data collection for this project were collaborative efforts between the University of Guam Marine Laboratory, Kosrae Conservation and Safety Office, Kosrae Department of Resources and Economic Affairs Division of Marine Resources, and Kosrae Island Resource Management Authority. These organizations are responsible for coral-reef monitoring in Kosrae and have the authority to conduct research. Further, non-invasive research was conducted using visual estimates described in the methods. Given the non-invasive nature of research, no permits were required.

### Study area

Kosrae is the easternmost of the Federated States of Micronesia (FSM), located 600 km north of the equator, approximately midway between Guam and Hawaii. Kosrae is a small (110 km^2^) high island covered in forested watersheds, lined with mangroves, and surrounded by a fringing outer reef [39]. Although the island has a small population (6,616) with little urban development (3% of total land area), human density per reef area is moderately high (130 individuals per km^2^ reef, approximately six times higher than FSM average), and the local population depends heavily on marine resources.

### Modern coral-reef assessments

**Fig 1:**
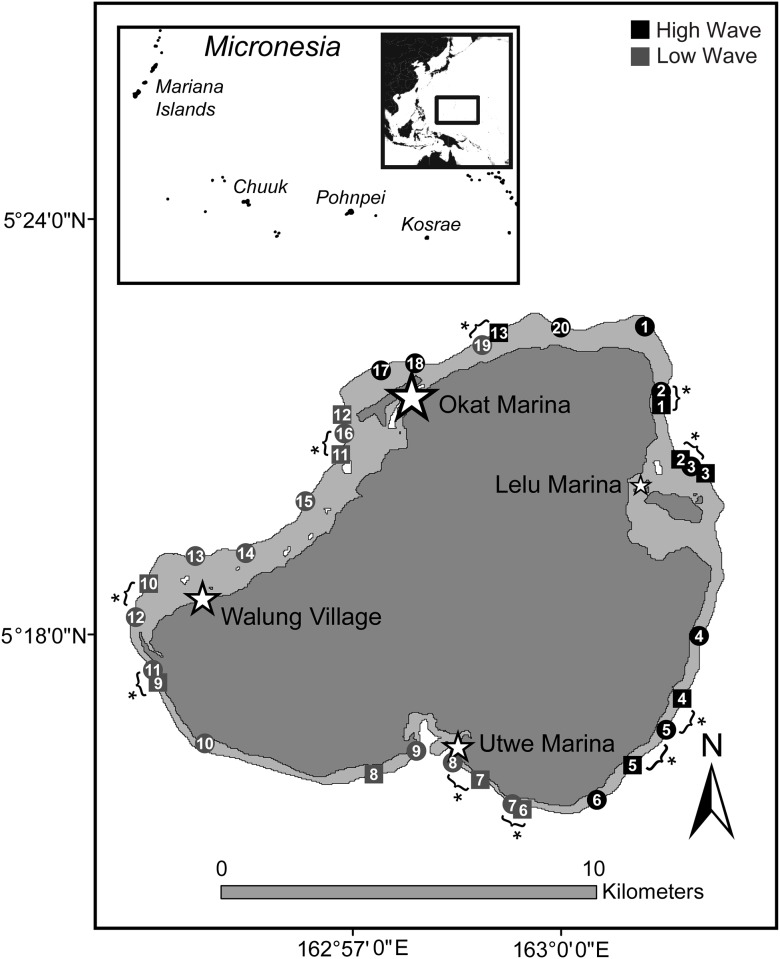
Map of Kosrae showing the location of sites surveyed in 1986 (circles) and 2015 (squares). The four major fishing marinas are indicated by stars, which are scaled by the number of boats housed within. Overlapping sites that were surveyed in both timeframes are indiated with brackets and asterisks. Open-access geographic information systems mapping layers were obtained from http://freegisdata.rtwilson.com.

Quantitative fish and coral composition data were collected from 13 sites around Kosrae, as part of a long-term coral-reef monitoring program (Fig 1). Sites were stratified based on geography, wave energy, watershed size, and human presence. During each survey, fishes and corals were assessed at the 8 m depth contour along five 50-m transects, following standardized protocols [40]. Protocols were designed to characterize reef slope habitats with high statistical power to detect both univariate and multivariate changes through time [40]. The 2015 monitoring sites were all within 0.3–1.3 km of estimated historical survey locations.

Fish observers entered the water first and conducted surveys while laying out transects. Fish surveys used twelve stationary point counts (SPC) spaced equally across the study area [41]. During each SPC, the observer recorded the species, abundances, and estimated sizes of all food-fishes within a 5-m radius, for 3 min. Food-fishes were defined as acanthurids, scarids, serranids, carangids, labrids, lethrinids, lutjanids, balistids, kyphosids, mullids, holocentrids, haemulids, and sharks (S1 Appendix). All size estimates were converted to biomass (kg) using length-weight coefficients from FishBase (www.fishbase.org) and fishery-dependent data collected across Micronesia (Cuetos-Bueno, unpublished data).

To account for disparity between SPC methods that are limited to a 5-m radius and historical surveys that had no limit, a second fish observer not using a distance boundary swam alongside the primary observer and recorded fish abundances for larger food-fishes that were less frequent within the 5-m radius. These included fishes such as lutjanids, carangids, and haemulids, but excluded smaller scarids and acanthurids (S1 Appendix).

Coral assemblages were assessed using standard 1-m^2^ quadrats placed at 25-m intervals along the transect lines (n = 10). Coral colonies whose center point was inside the quadrat were recorded to species level and measured across the widest diameter and the perpendicular diameter (S2 Appendix).

### Historical coral-reef assessments

Data from a 1986 Army Corps of Engineers survey of 20 reef slopes around Kosrae were digitized using appendices in the survey report [39]. Site locations were approximated from an atlas accompanying the survey report, and nine of these sites overlapped spatially with modern sites (Fig 1). During each historical survey, observed species were recorded and assigned to abundance categories. For corals these were: dominant (D), abundant (A), common (C), occasional (O), and rare (R). For fishes these were abundant (A), common (C), occasional (O), and rare (R). Because historical data were qualitative, no direct comparisons were made with modern data. Instead, we assessed (i) changes in fish and coral assemblages with respect to environmental conditions and local stressors in each time frame, and (ii) the difference in the relative ‘condition’ of each reef in both time frames. Both assessments required a standardized set of quantitative metrics, thus it was necessary to derive quantitative fish biomass and coral cover estimates from historical DACOR data (see supporting information S1 Text).

We assigned numerical fish abundances to each of the corresponding DACOR categories using median values from ranges provided in the historical report, and then converted abundances into biomass estimates. For corals, we used naturally-occurring groupings in modern coral cover data (%) to quantify the categorical abundances used in the 1986 survey. Coral cover values were assigned to DACOR categories using Jenk’s Natural Breaks Optimizations for coral cover distributions from islands across Micronesia. Jenk’s Natural Breaks Optimization is a data clustering method for defining natural groupings in continuous data (S1 Fig). Strikingly similar coral cover groupings were found across islands, and we therefore used the values found in Kosrae in 2015.

### Environmental factors

The environmental factors used in this analysis are well-documented drivers of fish and coral assemblage structure across Micronesia [35], including wave energy, watershed size, fishing access, and pollution. Wave energy values were generated for each site using a 10-year record of wind-speed, fetch distance, and angle of wind exposure (Quicksat wind data sets from 1999 to 2009; https://winds.jpl.nasa.gov), [35]. Watershed sizes (km^2^) were measured in ArcGIS using United States Geological Survey topographic maps.

A proxy for modern fishing access was derived by integrating local fishing access, boat-based fishing access, and wave energy. Local fishing access was calculated by multiplying the standardized values for: 1) the number of fishermen residing in the municipality adjacent to a site (2010 FSM Census, http://www.sboc.fm/), and 2) linear distance to the nearest access point or human residence. Boat-based fishing access was calculated by multiplying the standardized values for: 1) the number of fishing boats kept in each marina (Okat Marina, Lelu Marina, Utwe Marina, and Walung Village), and 2) the distance from each site to each marina. For both local and boat-based fishing access, distances were negatively scaled so that increasing distance yielded lower fishing access. Standardized values of local and boat-based fishing access were then multiplied by the proportion of days per year considered accessible for fishing (unfishable conditions defined as wind speeds > 6 m/s with fetch distances ≥ 20 km). A proxy for historical fishing access was similarly derived, but boat-based fishing access only considered the distance from each site to each marina, and local fishing access only considered the distance from each site to the nearest access point. We excluded the number of fishing boats kept in each marina, and the number of fishermen residing in adjacent municipalities because these data were unavailable for 1986.

A proxy for modern watershed pollution was calculated by multiplying standardized values of: 1) the total area of altered land in the adjacent watershed (barren, urban, or developed infrastructure), 2) human population in the adjacent watershed, and 3) the distance from a site to the nearest discharge point. As with fishing access, distances were negatively scaled so that increasing distance from discharge yielded lower pollution. Altered land area was derived using United States Forest Service land-use data (United States Forest Service, http://www.fs.usda.gov/r5). No historical proxy for pollution was used because data on altered land and watershed population were not available for 1986.

### Data analysis

#### Multivariate spatial composition

The spatial distribution of both fish and coral assemblages were assessed during each time frame using standard multivariate approaches [42]. Spatial differences were assessed with respect to wave energy, watershed size, fishing access, and pollution. In all instances, fish biomass and coral cover data were aggregated by functional group (e.g., large-bodied scarids, arborescent *Acropora*), log-transformed and Bray-Curtis dissimilarity coefficients were calculated between each pair of sites. Bray-Curtis dissimilarities were depicted using principal coordinate ordination (PCO) plots and the influences of environmental gradients and local stressors were determined using redundancy analysis. Significant environmental factors were overlaid on the PCO plots using vectors associated with each axis.

Initial results indicated that spatial differences in coral assemblages existed between high and low wave energy zones (i.e., windward and leeward sides of the island), with a higher degree of difference in 2015. Analyses of similarity (ANOSIM) were therefore used to determine if separation between high and low wave energy zones was greater in 2015 than 1986. Next, In order to determine if these findings were an artifact of different survey methods, SIMPER tests were used to determine which species contributed most to assemblage differences between high and low wave energy sites. Because spatially-restricted quadrat surveys were used in 2015, it is possible that larger differences between coral assemblages in high and low wave energy were an artifact of overlooked rare species. Therefore species with the largest contributions to SIMPER analyses were examined across wave energy conditions and the magnitude of differences were compared between time frames. For these analyses, sites with average wave energy values above 800 J/m3 (i.e., northeast exposure) were categorized as high, while sites lower than 800 J/m3 (i.e., southwest exposure) were categorized as low.

#### Latent variables and assemblage condition

**Fig 2:**
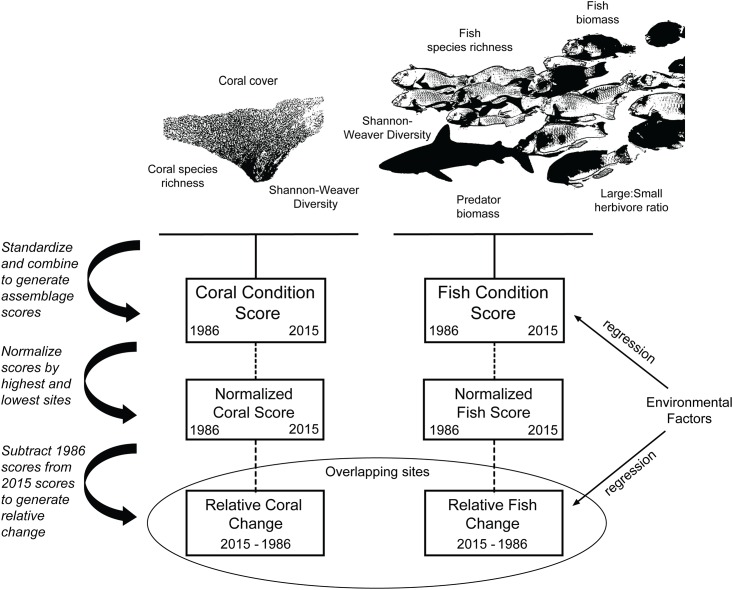
Depicition showing how individual metrics of fish and coral assemblages were combined to form latent variables of overall fish and coral condition, and how latent variables were used to test the influence of environmental factors on fish and coral changes through time.

We created latent variables to provide overall-condition scores for fish and coral assemblages during both time frames (Fig 2). Latent variables accounted for multiple factors that together determine resilience, and were less sensitive to small localized disturbances. For instance, Micronesia reefs with high coral cover often have low diversity, while those with lower cover, following disturbances, generally have higher diversity, and both components are important indicators of resilience [35] Ecological attributes used to generate fish latent variables were fish assemblage biomass, predator biomass, biomass ratios of large-to-small bodied herbivores, Shannon-Weaver diversity, and species richness. Large herbivore species were defined as those with estimated asymptotic lengths over 40 cm, while small herbivore species were defined as those with asymptotic lengths under 40 cm. These attributes were chosen by a regional working group of scientists to represent key ecological functions [35]. The individual attributes were standardized and averaged together to generate latent variables. The ecological attributes used to generate coral latent variables were coral cover, Shannon-Weaver diversity, and species richness. Sensitivity analyses were performed to determine which attributes were most influential to overall-condition scores, and to determine attribute covariance (Pearson’s correlations).

We examined the influence of wave energy, watershed size, fishing access, and pollution on the latent variables for fish and coral assemblages using a forward, stepwise regression modelling process. Synergistic interactions were only considered if they improved both the fit and stability of models. Resultant models were examined for residual normality using Shapiro-Wilk tests, and evaluated by their R^2^ values, p-values, and Akaike Information Criteria (AIC). One outlier, in the regression between historical fish assemblage condition and fishing access, was removed to meet the requirements of residual normality. Elsewhere, no outliers were found.

#### Validating modern fish surveys

To further examine potential bias from different fish survey protocols in 1986 and 2015, we compared key ecological attributes between modern fish observers, one using SPCs and a second mimicking the 1986 survey. Ecological attributes considered were fish-assemblage biomass, predator biomass, and Shannon-Weaver diversity. Other attributes were not compared because the secondary observer only surveyed a subset of the overall food fishes that are larger, heavily targeted, and potentially overlooked by modern protocols. Pearson’s moment correlations were used to determine the nature of these relationships.

#### Relative changes in fish and coral condition

We last examined the relative changes in fish and coral condition through time using the differences in normalized scores (i.e., 0–100) for spatially overlapping sites. Ten of the 2015 survey sites overlapped with a corresponding site from the 1986 survey (one site from 1986 was used twice), with estimated distances between 2015 and 1986 sites ranging between 0.1–1.3 km. The normalized fish and coral condition scores for the 1986 sites were subtracted from their 2015 counterparts, yielding relative change scores (S1 Table). Positive scores indicated that sites’ relative assemblage condition has increased through time, while negative scores indicated condition has declined. Correlations were used to determine if relative changes in fish and coral condition were spatially linked. Multiple regression modeling was used to determine if wave energy, watershed size, fishing access, or pollution could significantly predict the difference in normalized values.

## Results

### Multivariate spatial composition

**Fig 3:**
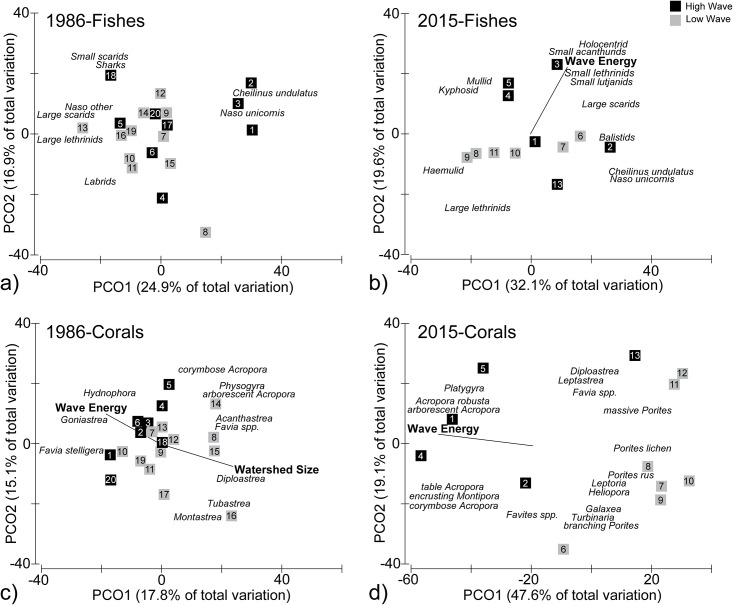
Principal component plots of fish (a, b) and coral (c, d) assemblage structure in 1986 and 2015. Major functional groups driving community structure are shown. Wave energy and watershed size were overlaid if they were significant predictors of assemblage structure. “Large” and “small” labels correspond to large-bodied and small-bodied groups refered to in the text.

**Fig 4:**
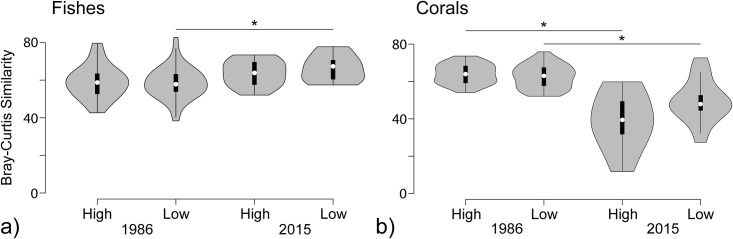
Violin plots showing the mean Bray-Curtis similarity values for fish (a) and coral (b) assemblages in high and low wave energy zones in 1986 and 2015. The white dots indicates the median, and the black bars indicate the interquartile range. Significant differences (p<0.05) are indicated with a single asterisk.

Fish assemblage structure was not predicted by either wave energy or watershed size in 1986 (P > 0.05, Redundancy Analyses; Fig 3a). In contrast, wave energy predicted fish assemblage structure in 2015 (DISTLM, Pseudo F = 2.1, P < 0.05; Fig 3b). High wave energy zones were distinguished by greater abundances of holocentrids, small-bodied acanthurids, small-bodied lethrinids, small-bodied lutjanids, and large-bodied scarids, while low wave energy zones were distinguished by greater abundances of large-bodied lethrinids and haemulids. These results suggested that fish assemblages were rather uniform across wave energy zones in 1986, but have become spatially differentiated over time. To better understand shifts in fish distributions, Bray-Curtis similarities were used to evaluate ecological similarity among sites within each timeframe and each wave energy zone. Bray-Curtis values were similar among sites within both high and low wave energy zones in 1986 and 2015, suggesting that wave energy has not changed assemblage homogeneity through time (Fig 4a). In contrast, Bray-Curtis values have become more homogenous in low wave energy zones in 2015 compared to 1986, but stayed the same within high wave energy zones (Kolmogorov-Smirnov tests, P < 0.05).

Expectedly, fish assemblage composition has shifted between 1986 and 2015, where many large-bodied predators and sharks have been replaced by smaller-bodied taxa in lower trophic levels (analysis of similarity contributions, Table 1). A large concern with these results was the influence of different survey methods. To address this, we report positive correlations between modern surveys using spatially-restricted SPC methods and spatially-unrestricted methods for fish assemblage biomass (r = 0.53), predator biomass (r = 0.54, r = 0.79 with one outlier removed), and Shannon-Weaver diversity (r = 0.41, r = 0.73 with one outlier removed). These findings suggested that spatial trends within each time frame were not necessarily an artifact of different survey methods, and declines in large predators and herbivores indicated shifting species abundance patterns through time.

**Table 1:** Fish functional groups contributing the top 50% of fish assemblage composition in high and low wave energy in 1986 and 2015. “Large” and “small” labels correspond to large-bodied and small-bodied groups refered to in the text.

Fish Assemblages							
High Wave Energy				Low Wave Energy			
1986		2015		1986		2015	
Taxa	Cumulative %	Taxa	Cumulative %	Taxa	Cumulative %	Taxa	Cumulative %
Large Lutjanid	13.33	Small acanthurid	19.07	Shark	15.41	Small scarid	23.75
Shark	26.20	Small Scarid	36.30	Large Lutjanid	30.46	Small acanthurid	44.56
Small Lutjanid	38.41			Small Lutjanid	40.43		
Naso lituratus	46.27			Large Scarid	49.19		

Both wave energy and watershed size predicted coral assemblage structure in 1986 (DISTLM, wave Pseudo F = 1.9, P < 0.05; watershed Pseudo F = 2.6, P < 0.005; Fig 3a). Wave energy and watershed size had a moderate, inverse relationship (r = 0.59, P < 0.05) suggesting the two factors did not necessarily act independently. Coral assemblages associated with high wave energy and small watersheds were characterized by species with encrusting and massive growth forms, including *Hydnophora*, *Goniastrea*, and *Favia stelligera*. Coral assemblages in low wave energy zones near large watersheds were characterized by corals commonly known to tolerate or benefit from sediments and nutrients including *Diploastrea* and *Tubastrea*. Coral assemblages in intermediate wave-energy zones or near intermediate-sized watersheds included a mixture of these corals (Fig 3a). Interestingly, *Acropora* were not characteristic of any single environmental regime in 1986, and were spatially consistent around Kosrae.

Wave energy alone predicted coral assemblage structure in 2015, with distinct assemblages in high and low wave energy zones (DISTLM, Pseudo F = 7.8, P < 0.05; Fig 3b). High wave energy zones were characterized by *Acropora* and *Montipora*, while low wave energy zones were characterized by *Porites* and *Galaxea*. Overall, wave energy was the only environmental factor driving coral assemblage structure during both time frames, and differences between high and low wave energy assemblages became more pronounced through time (Table 2). Bray-Curtis similarities among sites were uniform between high and low wave energy zones in both 1986 and 2015, but had reduced homogeneity in both areas in 2015 (Kolmogorov-Smirnov test, P < 0.05; Fig 4b).

**Table 2:** Coral functional groups contributing the top 50% of coral assemblage composition in high and low wave energy in 1986 and 2015.

Coral Assemblages							
High Wave Energy				Low Wave Energy			
1986		2015		1986		2015	
Taxa	Cumulative %	Taxa	Cumulative %	Taxa	Cumulative %	Taxa	Cumulative %
*Platygyra*	7.56	*Platygyra*	20.47	*Galaxea*	7.55	*Porites rus*	20.35
*Acropora* table	13.55	*Acropora* arborescent	37.29	*Acropora* Table	14.20	*Galaxea*	33.25
*Acropora robusta*	19.44	*Acropora robusta*	49.20	*Porites* massive	19.62	*Leptoria*	45.96
*Galaxea*	25.08			*Platygyra*	24.89		
*Pocillopora*	30.45			*Montipora* encrusting	29.92		
*Hydnophora*	35.77			*Porites rus*	34.82		
*Porites* massive	40.77			*Leptoria*	39.64		
*Monitpora* encrusting	45.71			*Acropora robusta*	44.26		
				*Favia*	48.43		

Increasing dissimilarity between coral assemblages in high and low wave energy did not appear to be biased by different survey methods. For example, arborescent *Acropora* were frequently reported as abundant and common on the leeward side of the island in 1986, while they were almost never encountered on the leeward side of the island in 2015. However, many other corals reported as common in 1986 were frequently encountered in 2015 (e.g., *Leptoria*, *Platygyra*, *Pavona*). Thus, the major difference between wave regimes in 2015 appeared to be an indication of shifting species assemblages through time.

### Latent variables and assemblage coition

Fishing access was the only significant predictor of fish assemblage condition in 1986, where increased fishing access was associated with higher fish assemblage condition (multiple regression modelling, R^2^ = 0.20, P = 0.05). Thus, commercially favorable assemblages existed on the leeward side of the island with greater fishing access. In contrast, there was a non-significant, negative association between fishing access and assemblage condition in 2015 (R^2^ = 0.18, P = 0.19) as condition values were either equal or better on the windward side. For corals, watershed size predicted overall condition in 1986, with higher condition near intermediate-sized watersheds (R^2^ = 0.43, P < 0.005). In contrast, the proxy to pollution was the only negative predictor of coral condition in 2015 (R^2^ = 0.36, P < 0.05), suggesting a potential shift from prevailing natural environmental factors to human stressors, similarly found for fish assemblages. Wave energy alone did not predict fish or coral latent variables in either time frame.

Sensitivity analysis further showed that fish species richness, Shannon-Weaver diversity, and fish assemblage biomass had the largest correlations to the latent variable for overall fish condition in 1986, indicating that these attributes were positively correlated and the most influential (r = 0.9, r = 0.82, and r = 0.79 respectively). Predator biomass and the ratio of large to small herbivores had lower and more unique contributions (r = 0.57 and r = 0.55 respectively). In contrast, predator biomass, Shannon-Weaver diversity, and species richness had the highest correlations with fish condition in 2015 (r = 0.79, 0.75, 0.74, respectively). Thus, declining predator abundance was a key component of shifting fish condition through time. Sensitivity analyses found that Shannon-Weaver diversity and species richness had strongest influences on the coral latent variable in both timeframes (r = 0.97 for both variables in 1986; r = 0.93 and 0.83 in 2015).

### Relative changes in fish and coral condition

**Fig 5:**
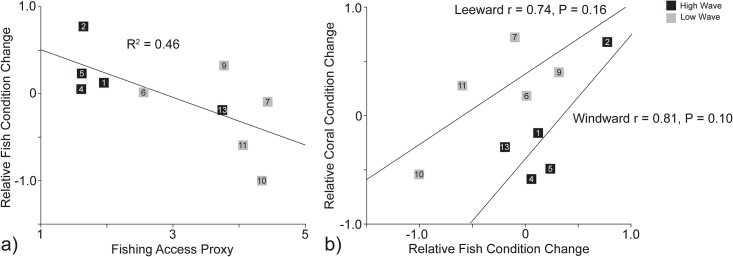
Linear regression depicting the influence of fishing access on the relative change in fish condition through time (a), and correlation depicting the relationship between the relative change in fish condition and the relative change in coral condition (b).

For overlapping sites surveyed in both timeframes, the relative changes in fish and coral condition were associated, but these associations differed with wave energy. Fish changes appeared more pronounced than coral changes on the leeward side, while coral changes appeared more pronounced than fish changes on the windward side (Pearson’s moment correlations, leeward: n = 5 sites, r = 0.74, P = 0.16, windward: n = 5 sites, r = 0.8, P = 0.10; Fig 5b). More notably, fishing access predicted declining fish condition through time (multiple regression modelling, P < 0.05; Fig 5a). Changes in coral condition were not predicted by any environmental factor through time, but noted above, were associated with changes in fish condition.

## Discussion

We report distributions of fish and coral assemblages around Kosrae that were historically more heterogeneous, and influenced by natural environments, but have become increasingly distinctive and influenced by local stressors. In 1986, fish assemblages were similar around the island, and dominated by large predators such as *Carcharhinus amblyrhynchos* and *Lutjanus bohar*, and large herbivores such as *Acanthurus blochii* and *Scarus altipinnis*. Both spatially-restricted and unrestricted surveys now show significant differences in fish assemblages between windward and leeward areas, few predators and large herbivores, and composition shifting to smaller, site-attached herbivores and secondary consumers like *Ctenochaetus spp*., *Chlorurus sordidus*, *Lutjanus fulvus*, and *Monotaxis grandoculis*. In contrast, coral assemblages were distinct in windward and leeward areas in 1986, but with much overlap in species composition, and well-mixed, diverse reefs along the northern and southern coastline where wave energy transitions. However, in 2015, windward and leeward areas had become highly differentiated with very little species overlap. Windward sites were dominated by fast-growing arborescent *Acropora* and other corals that are common to oligotrophic waters with high wave energy, while leeward sites were dominated by slow-growing, massive corals that can tolerate turbid, sediment-rich water and derive more energy from heterotrophic feeding [43,44].

In 1986, fish condition was highest in leeward, wave-protected areas. Leeward areas had larger watersheds with greater terrestrial input, larger reef flats and lagoons, gently sloping fore-reefs, and lower wave action and flushing, altogether providing higher productivity and more habitat for fisheries [45–49]. While fish assemblages were more commercially valuable on the leeward side of the island, fishing access was also higher due to lower wave energy and higher human populations. Increased fishing pressure from a growing artisanal fishery appears to be shifting the distribution of favorable fish assemblages around the island, as fishes in windward sites were now in better condition. In contrast, coral condition declined more in windward sites. This may be partially influenced by localized pollution at one or two densely populated areas, and the absence of lagoons to dilute watershed discharge, however pollution was not identified as a long term driver of change. Although fish declines were higher in leeward areas, and coral declines were higher in windward areas, when considering windward and leeward areas separately, it appeared that changes in fish and coral condition were spatially linked—correlation coefficients strongly increased when stratifying by wave exposure. Overall, fishing access predicted fish assemblage decline through time, and declining fish condition was the only factor correlated with declining coral condition, providing evidence that fishing pressure may have indirectly reduced coral condition.

Based on our results, it appears that removal of key functional groups, particularly predators and large herbivores, reduced ecosystem resilience and led to reef decline through time. Overfishing coral reefs has repeatedly been shown to shift assemblages from dominance by large predators and herbivores to smaller species of herbivores and secondary consumers, reducing ecosystem function [36,50–52]. Larger fishes provide greater ecological function and are crucial for ecosystem maintenance; large herbivores open substrate for coral recruitment and limit algae overgrowth, while large predators stabilize food webs and promote diversity through top-down control [53–57]. The removal of large predators and herbivores therefore compromises these processes that regulate food web structure and stability [58,59]. Additionally, assemblages dominated by smaller fishes lack metapopulation connectivity and may fail to re-seed exploited populations in distant areas [60–62].

Still the question remains, how did coral communities change so dramatically, especially considering that *Acropora* corals were common in leeward areas in 1986, but are now almost entirely absent? Previous articles, reports, and personal communications indicate that large-scale disturbances have been minimal in Kosrae, with no major bleaching events, crown of thorns starfish outbreaks, or typhoons documented between 1986 and 2015 [63]. This is intriguing because other studies have noted declining coral condition in areas with exploited fisheries, but typically after a major disturbance reduces living coral cover [11,52]. We postulate that small, localized disturbances may have contributed to a slow and gradual shifting baseline. For instance, anecdotal reports from resource managers and tourist operators revealed that storm surges in 2008 and 2015 caused isolated damage to reefs. Additionally, mild thermal stress in 2013 caused isolated damage to one reef in the south (personal observation). When key ecosystem functions are compromised, reefs become vulnerable to small incremental damage from disturbances like these [64–66].

### Conclusions

Historical baselines are rare for coral reefs, and often require creative approaches to compare them with modern data [23,67]. While rare, such baselines provide a fundamental source for assessing long-term coral-reef dynamics, which offers key insights for resilience management. Comparing historical baseline data with modern surveys in Kosrae indicated that fishing access was the most influential local stressor causing change in reef assemblages. Further, we found that, even in the absence of large disturbances, local stressors can have substantial impacts when coupled with small-scale disturbances over time. Predator biomass and herbivore size were the most sensitive aspects of fish assemblage condition in 2015, followed by species richness, and declines in these attributes appeared to drive overall fish decline. Therefore, ecosystem-based fisheries management should focus on the restoration and maintenance of large predators and herbivores. Modern site #2 provides a good example as it was severely damaged by a storm surge in 2008 (anecdotal reports, personal observations), but only seven years later holds the highest fish and coral species richness, along with the highest large-herbivore biomass and second-highest predator biomass. This appears to be a promising example of how intact, healthy fish assemblages can help reefs recover after small disturbances. Managing other areas to capture this resilience is key to protecting Kosrae’s reefs into the future. While strictly impeding fish harvest would damage the economy and livelihood of Kosrae’s people, sound fisheries management can reduce fishing impacts through seasonal species bans, catch quotas, and size windows. Restructuring fish harvest can help create sustainable fisheries with increased functionality, which will strengthen resilience and mitigate coral-reef decline while maintaining food security.

## Supporting Information

S1 Appendix Fish species recorded in each of the fish surveys. 2015a refers to the stationary point count method, while 2015b refers to the spatially unrestricted method.
(DOCX)

S2 Appendix Coral species recorded in each of the coral surveys. (DOCX)

S1 Fig Boxplots showing the range of coral cover cut-off values from Jenk's Natural Breaks Optimization for four islands. Six cut offs were generated to create end points for five individual classes, representing the range of values between cut offs. Similar, power-law relationships were found for each island, indicating the inherent nature of coral abundance categories throughout Micronesia.
(TIF)

S2 Fig Estimated values of coral cover for the five coral abundance classes across Micronesia. Coral cover categories represent means between Jenk’s Breaks cut offs.
(TIF)

S1 Table Normalized fish and coral condition values for overlapping sites in 1986 and 2015, with relative condition changes. (DOCX)

S2 Table Environmental factors used for data analysis in 1986. (DOCX)

S3 Table Environmental factors used for data analysis in 2015. (DOCX)

S1 Text Supplementary information explaining the full methodology used to quantify the 1986 historical datasets. (DOCX)
