# Supplementary material for: Local Stressors, Resilience, and Shifting Baselines on Coral Reefs
Source: PLoS One. 2016 Nov 30;11(11):e0166319. doi: 10.1371/journal.pone.0166319 (PMC5130202; doi:10.1371/journal.pone.0166319)
Supplement: S1 Appendix — 2015a refers to the stationary point count method, while 2015b refers to the spatially unrestricted method. (DOCX) [file pone.0166319.s001.docx]

| Species | 1986 | 2015a | 2015b |
| --- | --- | --- | --- |
| *Acanthurus blochii* | X | X | X |
| *Acanthurus guttatus* | X | X |  |
| *Acanthurus leucocheilus* |  | X |  |
| *Acanthurus lineatus* | X | X |  |
| *Acanthurus nigricans* | X | X |  |
| *Acanthurus nigrofuscus* | X |  |  |
| *Acanthurus olivaceous* | X |  |  |
| *Acanthurus pyroferus* | X | X |  |
| *Acanthurus triostegus* | X |  |  |
| *Acanthurus xanthopterus* | X | X | X |
| *Aethaloperca rogaa* | X |  |  |
| *Anyperodon leucogrammicus* | X |  |  |
| *Aphareus furca* | X | X | X |
| *Aprion viriscens* | X | X | X |
| *Balistoides viridescens* |  | X |  |
| *Bolbometopon muricatum* | X |  |  |
| *Calotomus carolinus* |  | X |  |
| *Carangoides ferdau* |  | X | X |
| *Caranx ignobilis* |  |  | X |
| *Caranx melampygus* | X | X | X |
| *Caranx sexfasciatus* | X |  |  |
| *Caranx sp.* | X |  |  |
| *Carcharhinus amblyrhynchos* | X |  |  |
| *Cephalopholis argus* | X | X | X |
| *Cephalopholis urodeta* | X | X |  |
| *Cetoscarus bicolor* | X |  |  |
| *Cheilinus chlorourus* | X |  |  |
| *Cheilinus fasciatus* | X | X |  |
| *Cheilinus trilobatus* | X | X |  |
| *Cheilinus undulatus* | X | X | X |
| *Chlorurus frontalis* |  | X | X |
| *Chlorurus japanensis* |  | X | X |
| *Chlorurus microrhinos* | X | X | X |
| *Chlorurus sordidus* | X | X |  |
| *Ctenochaetus striatus* | X | X |  |
| *Decapterus sp.* | X |  |  |
| *Epibulus insidiator* | X | X | X |
| *Epinephelus fuscoguttatus* | X |  |  |
| *Epinephelus merra* | X |  |  |
| *Epinephelus polyphekadion* | X |  |  |
| *Epinephelus sp.* | X |  |  |
| *Gymnosarda unicolor* | X |  |  |
| *Hipposcarus longiceps* | X | X | X |
| *Kyphosus bigibbus* |  |  | X |
| *Kyphosus cinerascens* | X | X |  |
| *Kyphosus sp.* | X |  |  |
| *Kyphosus vaigiensis* | X | X | X |
| *Lethrinus harak* | X | X |  |
| *Lethrinus microdon* | X |  |  |
| *Lethrinus miniatus* | X |  |  |
| *Lethrinus obsoletus* |  |  | X |
| *Lethrinus olivaceus* |  | X | X |
| *Lethrinus xanthochilus* |  | X | X |
| *Lethrinus sp.* | X |  |  |
| *Lutjanus argentimaculatus* |  | X | X |
| *Lutjanus bohar* | X | X | X |
| *Lutjanus fulviflamma* | X |  |  |
| *Lutjanus fulvus* | X | X | X |
| *Lutjanus gibbus* | X | X | X |
| *Lutjanus kasmira* | X |  |  |
| *Lutjanus monostigma* | X |  | X |
| *Lethrinus semicinctus* |  | X | X |
| *Lutjanus sp.* | X |  |  |
| *Macolor macularis* |  | X | X |
| *Macolor niger* | X | X | X |
| *Monotaxis grandoculis* | X | X | X |
| *Mulloidichthys flavolineatus* | X | X |  |
| *Mulloidichthys vanicolensis* |  | X |  |
| *Myripristis adusta* | X |  |  |
| *Myripristis berndti* | X |  |  |
| *Myripristis murdjan* | X |  |  |
| *Myripristis sp.* | X | X |  |
| *Myripristis violacea* | X |  |  |
| *Naso brevirostris* | X | X |  |
| *Naso hexacanthus* | X |  |  |
| *Naso lituratus* | X | X | X |
| *Naso thynnoides* | X |  |  |
| *Naso unicornis* | X | X | X |
| *Naso vlamingii* | X | X | X |
| *Neoniphon sammara* | X |  |  |
| *Neoniphon sp.* | X | X |  |
| *Parupeneus barberinus* | X | X |  |
| *Parupeneus bifasciatus* | X | X | X |
| *Parupeneus cyclostomus* | X | X |  |
| *Parupeneus indicus* | X |  |  |
| *Parupeneus multifasciatus* | X | X |  |
| *Pempheris oualensis* | X | X |  |
| *Platax orbicularis* | X |  |  |
| *Plectorhinchus albovittatus* |  | X | X |
| *Plectorhinchus lineatus* |  | X | X |
| *Plectorhinchus nigrus* | X |  |  |
| *Plectorhinchus pictus* | X |  |  |
| *Pseudobalistes flavimarginatus* |  | X |  |
| *Pygoplites diacanthus* | X | X |  |
| *Sargocentron caudimaculatum* | X |  |  |
| *Sargocentron spinniferum* | X |  |  |
| *Sargocentron tiere* | X | X |  |
| *Scarus altipinnis* | X | X | X |
| *Scarus dimidiatus* | X | X |  |
| *Scarus festivus* |  | X |  |
| *Scarus forsteni* |  | X |  |
| *Scarus frenatus* | X | X | X |
| *Scarus ghobban* | X | X | X |
| *Scarus globiceps* | X | X |  |
| *Scarus hypselopterus* | X |  |  |
| *Scarus niger* | X | X | X |
| *Scarus oviceps* | X | X |  |
| *Scarus ovifrons* | X |  |  |
| *Scarus prasiognathus* | X |  |  |
| *Scarus psittacus* | X | X |  |
| *Scarus rivulatus* | X | X | X |
| *Scarus rubroviolaceus* | X | X | X |
| *Scarus schlegeli* | X | X |  |
| *Scarus sp.* | X |  |  |
| *Scarus spinus* | X | X |  |
| *Scarus tricolor* | X |  |  |
| *Siganus argenteus* |  | X | X |
| *Siganus doliatus* |  | X | X |
| *Siganus puellus* | X |  |  |
| *Siganus virgatus* | X |  |  |
| *Sphyraena barracuda* | X |  |  |
| *Trachinotus blochii* | X |  |  |
| *Triaenodon obesus* | X |  |  |
| *Uindentified Mullid* | X |  |  |
| *Unidentified carangid* | X |  |  |
| *Unidentified Lutjanid* | X |  |  |
| *Unidentified Scarid* | X |  |  |
