# Supplementary material for: Local Stressors, Resilience, and Shifting Baselines on Coral Reefs
Source: PLoS One. 2016 Nov 30;11(11):e0166319. doi: 10.1371/journal.pone.0166319 (PMC5130202; doi:10.1371/journal.pone.0166319)
Supplement: S2 Appendix — (DOCX) [file pone.0166319.s002.docx]

| Species | 1986 | 2015 |
| --- | --- | --- |
| *Acanthastrea echinata* | X |  |
| *Acropora abrotanoides* |  | X |
| *Acropora aculeus* |  | X |
| *Acropora acuminata* | X |  |
| *Acropora austera* |  | X |
| *Acropora cerealis* | X | X |
| *Acropora cytherea* | X | X |
| *Acropora delicatula* | X |  |
| *Acropora digitifera* | X |  |
| *Acropora echinata* | X |  |
| *Acropora florida* | X | X |
| *Acropora formosa* | X |  |
| *Acropora gemmifera* | X |  |
| *Acropora hebes* | X |  |
| *Acropora humilis* | X |  |
| *Acropora hyacinthus* | X | X |
| *Acropora irregularis* | X | X |
| *Acropora macrostoma* |  | X |
| *Acropora microclados* |  | X |
| *Acropora monticulosa* | X |  |
| *Acropora nasuta* | X | X |
| *Acropora polystoma* |  | X |
| *Acropora robusta* |  | X |
| *Acropora squarrosa* | X |  |
| *Acropora syringodes* | X |  |
| *Acropora tenuis* |  | X |
| *Acropora valida* | X |  |
| *Acropora variabilis* | X |  |
| *Acropora vaughani* | X |  |
| *Alveopora allingi* | X |  |
| *Alveopora verrilliana* | X |  |
| *Barabattoia amicorum* | X |  |
| *Coscinaraea columna* | X |  |
| *Cyphastrea microphthalma* | X | X |
| *Cyphastrea serailea* | X | X |
| *Danafungia repanda* | X |  |
| *Diploastrea heliopora* | X | X |
| *Distichopora violacea* | X |  |
| *Echinophyllia aspera* | X |  |
| *Echinopora gemmacaea* |  | X |
| *Echinopora lamellosa* | X | X |
| *Euphyllia cristata* | X |  |
| *Favia favus* | X |  |
| *Favia laxa* | X |  |
| *Favia matthaii* | X | X |
| *Favia pallida* | X |  |
| *Favia rotundata* | X |  |
| *Favia stelligera* | X | X |
| *Favites abdita* | X | X |
| *Favites complanata* |  | X |
| *Favites flexuosa* | X |  |
| *Favites halicora* | X | X |
| *Favites pentagonia* | X | X |
| *Favites russelli* | X | X |
| *Fungia fungites* | X | X |
| *Fungia repanda* |  | X |
| *Fungia scutaria* |  | X |
| *Galaxea astreata* |  | X |
| *Galaxea fasicularis* | X | X |
| *Gardineroseris planulata* | X |  |
| *Goniastrea pectinata* | X | X |
| *Goniastrea retiformis* | X | X |
| *Goniopora columna* | X | X |
| *Goniopora djiboutiensis* | X |  |
| *Goniopora fruticosa* |  | X |
| *Goniopora lobata* | X |  |
| *Goniopora minor* |  | X |
| *Goniopora somaliensis* | X |  |
| *Halomitra pileus* | X |  |
| *Heliopora coerulea* | X | X |
| *Hydnophora exesa* | X |  |
| *Hydnophora microconos* | X | X |
| *Hydnophora rigida* | X | X |
| *Isopora cuneata* | X |  |
| *Isopora palifera* | X |  |
| *Leptastrea bottae* | X |  |
| *Leptastrea purpurea* | X | X |
| *Leptastrea transversa* | X |  |
| *Leptoria phrygia* | X | X |
| *Leptoseris explanata* | X |  |
| *Leptoseris incrustans* |  | X |
| *Leptoseris mycetoseroides* | X |  |
| *Leptoseris scabra* | X |  |
| *Lobophyllia corymbosa* | X |  |
| *Lobophyllia hemprichii* | X | X |
| *Merulina ampliata* | X |  |
| *Millepora dichotoma* | X |  |
| *Millepora exaesa* | X |  |
| *Millepora platyphylla* | X |  |
| *Millepora tuberosa* |  | X |
| *Montastrea curta* | X | X |
| *Montipora caliculata* | X | X |
| *Montipora digitata* | X |  |
| *Montipora efflorescens* |  | X |
| *Montipora ehrenbergii* | X |  |
| *Montipora floweri* | X | X |
| *Montipora foliosa* | X |  |
| *Montipora foveolata* | X |  |
| *Montipora granulosa* | X |  |
| *Montipora grisea* |  | X |
| *Montipora hispida* | X |  |
| *Montipora hoffmeisteri* | X | X |
| *Montipora informis* |  | X |
| *Montipora lobulata* |  | X |
| *Montipora marshallensis* | X |  |
| *Montipora monasteriata* | X | X |
| *Montipora patula* |  | X |
| *Montipora tuberculosa* | X | X |
| *Montipora turgescens* |  | X |
| *Montipora verrilli* | X |  |
| *Montipora verrucosa* | X | X |
| *Mycedium elephanototus* | X |  |
| *Oulophyllia crispa* | X |  |
| *Oxypora glabra* |  | X |
| *Oxypora lacera* | X | X |
| *Pachyseris rugosa* | X |  |
| *Pachyseris speciosa* | X |  |
| *Pavona clavus* | X |  |
| *Pavona divaricata* | X |  |
| *Pavona duerdeni* |  | X |
| *Pavona explanulata* | X |  |
| *Pavona varians* | X | X |
| *Physogyra lichtensteinii* | X | X |
| *Platygyra daedalea* | X | X |
| *Platygyra lamellina* | X |  |
| *Platygyra pini* | X | X |
| *Plerogyra sinuosa* | X |  |
| *Plesiastrea versipora* | X |  |
| *Pleuractis paumotensis* | X |  |
| *Pleuractis scutaria* | X |  |
| *Pocillopora ankeli* |  | X |
| *Pocillopora brevicornis* | X |  |
| *Pocillopora damicornis* | X |  |
| *Pocillopora elegans* |  | X |
| *Pocillopora eydouxi* | X |  |
| *Pocillopora meandrina* | X | X |
| *Pocillopora verrucosa* | X | X |
| *Porites attenuata* |  | X |
| *Porites australiensis* | X | X |
| *Porites cylindrica* | X | X |
| *Porites lichen* | X | X |
| *Porites lobata* | X | X |
| *Porites lutea* | X | X |
| *Porites murrayensis* | X |  |
| *Porites rus* | X | X |
| *Porites solida* | X | X |
| *Porites nigrescens* | X |  |
| *Porites vaughani* |  | X |
| *Psammacora contigua* | X |  |
| *Psammacora digitata* | X |  |
| *Psammacora nierstraszi* | X | X |
| *Sandalolitha robusta* | X |  |
| *Stylocoeniella armata* | X | X |
| *Stylocoeniella guntheri* | X | X |
| *Symphyllia recta* | X |  |
| *Symphyllia valenciennesii* | X |  |
| *Tubastrea coccinea* | X |  |
| *Tubipora musica* | X |  |
| *Turbinaria reniformis* |  | X |
| *Turbinaria stellulata* | X | X |
| *Verrillofungia concinna* | X |  |
