# Supplementary figures and images for: Local Stressors, Resilience, and Shifting Baselines on Coral Reefs
Source: PLoS One. 2016 Nov 30;11(11):e0166319. doi: 10.1371/journal.pone.0166319 (PMC5130202; doi:10.1371/journal.pone.0166319)

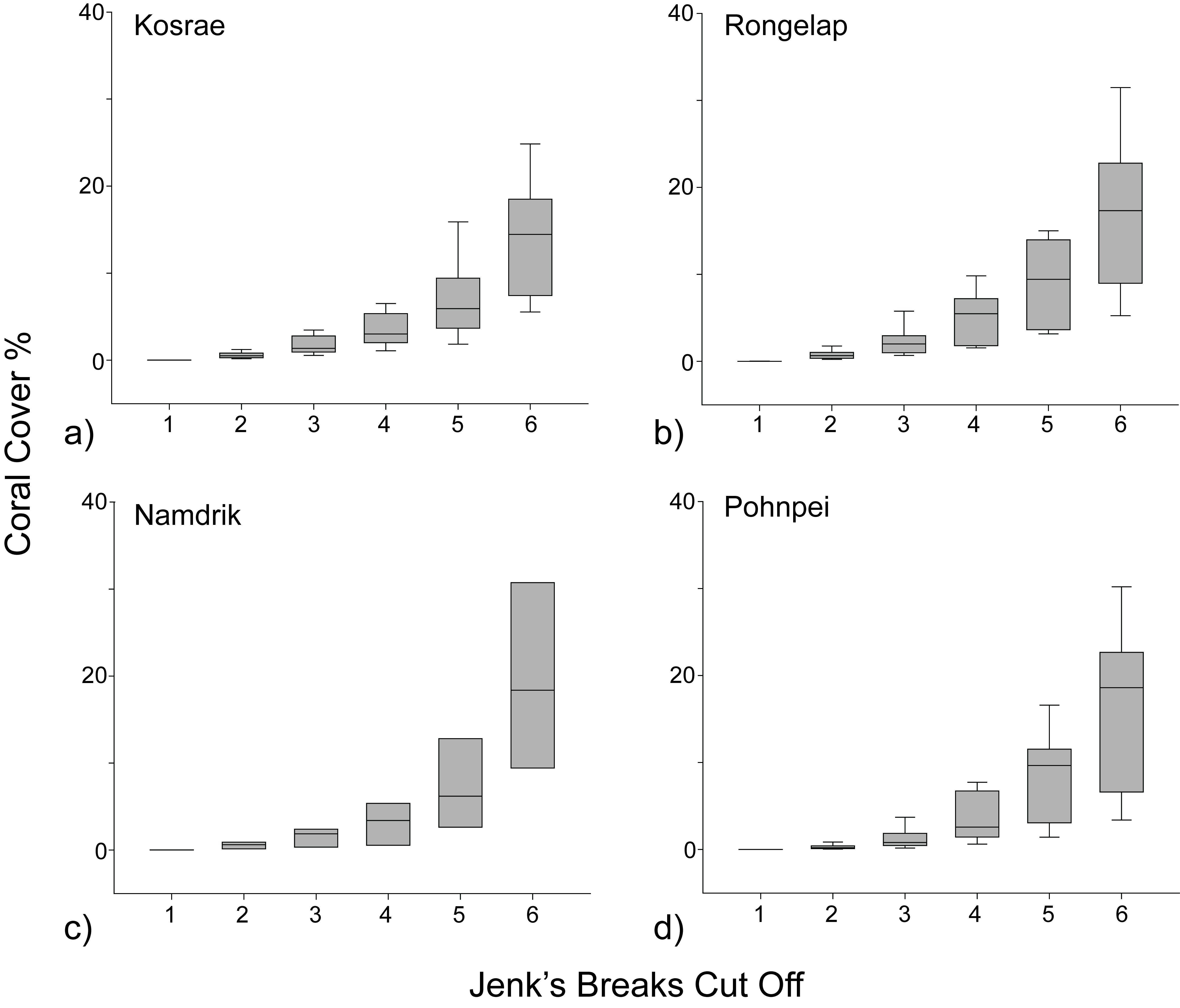

Supplement: S1 Fig — Six cut offs were generated to create end points for five individual classes, representing the range of values between cut offs. Similar, power-law relationships were found for each island, indicating the inherent nature of coral abundance categories throughout Micronesia. (TIF) [file pone.0166319.s003.tif]

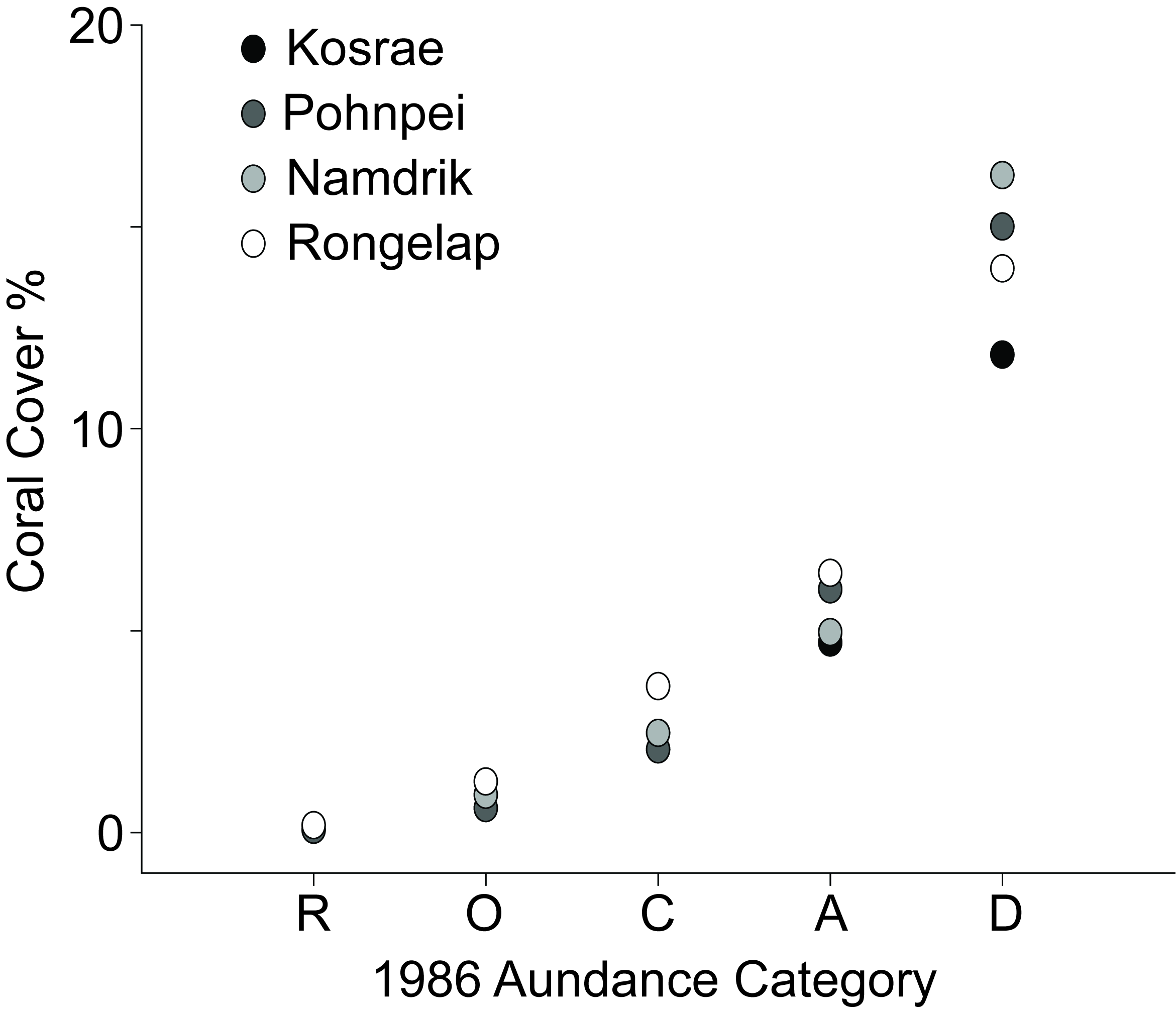

Supplement: S2 Fig — Coral cover categories represent means between Jenk’s Breaks cut offs. (TIF) [file pone.0166319.s004.tif]
