# Supplementary material for: Local Stressors, Resilience, and Shifting Baselines on Coral Reefs
Source: PLoS One. 2016 Nov 30;11(11):e0166319. doi: 10.1371/journal.pone.0166319 (PMC5130202; doi:10.1371/journal.pone.0166319)
Supplement: S1 Table — (DOCX) [file pone.0166319.s005.docx]

| 1986 Site # | 2015  Site # | 1986 Fish Condition Score | 2015 Fish Condition Score | Fish  Condition  Change | 1986 Coral Condition | 2015 Coral Condition | Coral Condition Change |
| --- | --- | --- | --- | --- | --- | --- | --- |
| 2 | 1 | 0.21 | 0.34 | 0.13 | 0.62 | 0.46 | -0.16 |
| 3 | 2 | 0.23 | 1 | 0.77 | 0.33 | 1 | 0.67 |
| 5 | 4 | 0 | 0.05 | 0.05 | 0.59 | 0 | -0.59 |
| 5 | 5 | 0 | 0.23 | 0.23 | 0.59 | 0.09 | -0.50 |
| 7 | 6 | 0.38 | 0.40 | 0.01 | 0.29 | 0.47 | 0.18 |
| 8 | 7 | 0.13 | 0.04 | -0.09 | 0 | 0.72 | 0.72 |
| 11 | 9 | 0.30 | 0.63 | 0.32 | 0.30 | 0.70 | 0.40 |
| 12 | 10 | 1 | 0 | -1 | 1 | 0.47 | -0.53 |
| 16 | 11 | 0.67 | 0.08 | -0.59 | 0.06 | 0.33 | 0.28 |
| 19 | 13 | 0.84 | 0.65 | -0.19 | 0.52 | 0.40 | -0.29 |
