# Supplementary material for: Local Stressors, Resilience, and Shifting Baselines on Coral Reefs
Source: PLoS One. 2016 Nov 30;11(11):e0166319. doi: 10.1371/journal.pone.0166319 (PMC5130202; doi:10.1371/journal.pone.0166319)
Supplement: S2 Table — (DOCX) [file pone.0166319.s006.docx]

| 1986 Site # | Wave Energy (J/m3) | Watershed  Size (km²) | Fishing Proxy |
| --- | --- | --- | --- |
| 1 | 2556.68 | 2.69 | 1.63 |
| 2 | 2383.54 | 3.83 | 1.90 |
| 3 | 2363.12 | 3.78 | 1.72 |
| 4 | 2361.68 | 2.34 | 1.13 |
| 5 | 2378.43 | 4.92 | 1.52 |
| 6 | 2396.34 | 4.67 | 1.55 |
| 7 | 609.68 | 4.67 | 2.91 |
| 8 | 91.24 | 13.76 | 5.59 |
| 9 | 54.92 | 9.51 | 2.07 |
| 10 | 231.28 | 6.30 | 2.58 |
| 11 | 153.81 | 4.43 | 3.28 |
| 12 | 176.03 | 4.43 | 4.07 |
| 13 | 141.07 | 6.37 | 3.92 |
| 14 | 128.48 | 6.37 | 4.43 |
| 15 | 126.66 | 8.47 | 3.90 |
| 16 | 129.43 | 14.28 | 4.53 |
| 17 | 968.35 | 2.97 | 3.65 |
| 18 | 1159.42 | 2.97 | 4.10 |
| 19 | 422.75 | 3.89 | 5.07 |
| 20 | 2322.79 | 2.69 | 1.95 |
