# Supplementary material for: Local Stressors, Resilience, and Shifting Baselines on Coral Reefs
Source: PLoS One. 2016 Nov 30;11(11):e0166319. doi: 10.1371/journal.pone.0166319 (PMC5130202; doi:10.1371/journal.pone.0166319)
Supplement: S3 Table — (DOCX) [file pone.0166319.s007.docx]

| 2015  Site # | GPS Y | GPS X | Wave Energy (J/m3) | Watershed Size (km²) | Fishing Proxy | Pollution Proxy |
| --- | --- | --- | --- | --- | --- | --- |
| 1 | 592400 | 280990 | 2372.30 | 3.83 | 1.95 | 8.65 |
| 2 | 590883 | 281512 | 2388.20 | 3.78 | 1.64 | 0 |
| 3 | 590465 | 282179 | 2387.40 | 3.78 | 1.74 | 0 |
| 4 | 584519 | 281554 | 2382.20 | 4.92 | 1.61 | 17.48 |
| 5 | 582746 | 280232 | 2350.74 | 3.31 | 1.62 | 7.00 |
| 6 | 581642 | 277193 | 788.60 | 4.67 | 2.55 | 9.54 |
| 7 | 582358 | 276178 | 127.50 | 3.35 | 4.43 | 8.42 |
| 8 | 582503 | 273344 | 239.80 | 4.76 | 2.25 | 5.57 |
| 9 | 585054 | 267521 | 156.80 | 4.43 | 3.77 | 4.68 |
| 10 | 587580 | 267346 | 200.40 | 4.43 | 4.34 | 4.10 |
| 11 | 591012 | 272464 | 122 | 14.27 | 4.10 | 6.56 |
| 12 | 592076 | 272499 | 131.73 | 14.27 | 3.87 | 4.12 |
| 13 | 594250 | 276670 | 813 | 3.89 | 3.74 | 21.58 |
