# Supplementary material for: Local Stressors, Resilience, and Shifting Baselines on Coral Reefs
Source: PLoS One. 2016 Nov 30;11(11):e0166319. doi: 10.1371/journal.pone.0166319 (PMC5130202; doi:10.1371/journal.pone.0166319)
Supplement: S1 Text — (DOCX) [file pone.0166319.s008.docx]

## **Supporting Information Methods**

## **Quantifying DACOR data:**

### **Corals**

In order to assign abundance values to DACOR datasets, we examined natural breaks in coral abundances from modern data for Kosrae and three nearby islands: Rongelap Atoll, Namdrik Atoll, and Pohnpei. Modern datasets were used to determine consistent, natural breaks in coral abundances approximating five DACOR categories. This approach provided an objective means to estimate Kosrae’s historical coral abundances in a categorical manner, reflecting the 1986 protocol.

Expert observers in 1986 used DACOR categories to estimate the total coverage of coral species with no dependence on colony size. This was evidenced by equal DACOR rankings for corals that attain large colony sizes but are few in number (e.g., *Acropora hyacinthus*) and corals that have small colony-sizes, but are prolific on reefs across Micronesia (e.g., *Leptastrea purpurea*). Jenks’ Natural Breaks Optimization (hereafter referred to as Jenks’ Breaks) was used to determine natural abundance categories for coral species from Kosrae, Rongelap Atoll, Namdrik Atoll, and Pohnpei. In all cases, coral abundance data were collected using the modern quadrat method. Jenk’s Breaks clustered coral-species abundance data into five categories (matching DACOR) by minimizing intra-class variation while maximizing inter-class variation (Jenks 1967). This process revealed a similar, exponential relationship between species coral cover and Jenk’s Breaks for all islands (S1 Fig.). We determined the values for D, A, C, O, and R by taking the mean coral cover between sequential breaks, D= 11.83%, A = 4.70%, C = 2.40%, O = 1.0%, R = 0.16% (S2 Fig.). We assigned these values to the 1986 coral dataset and compiled coral cover data at the species level and functional group level (e.g., branching *Acropora* or massive *Porites*) for data analyses. No direct temporal analyses were conducted using the assigned coverage values, rather coverage was used to examine spatial gradients in biological metrics. To ensure validity, initial multivariate plots of species abundance distributions were compared using these estimated coral cover values and rank order abundance values. Differences were non-significant, however rank abundance data diminished multivariate separation between contrasting coral assemblages, due to reduced cover estimates of prolific coral species.

### **Fishes**

For both 1986 and modern fish datasets, rare encounters of species with extremely high numeric densities were removed from the present analyses because they disproportionately influence overall composition and represent species that are not site-specific (S1 Appendix). During the 1986 surveys, fish abundances for each site were reported in the following ranges (number of fishes): abundant (A) = 15 or more, common (C) = 5-14, occasional (O) = 2-4, and rare (R) = 1. We defined an upper cut-off value for the abundant category using the maximum density observed in fish surveys conducted over similar areas from modern data across Micronesia (44 fishes); therefore A = 15-44. In order to examine the sensitivity of our fish abundance categories, initial multivariate comparisons were conducted using the minimum, maximum, and median values for each abundance category. Initial tests were also conducted using rank order abundance. In all instances, non-significant differences in spatial patterns were observed, with the similar finding that rank-order abundance diminished the magnitude of multivariate separation. Given non-significant differences, we logically used median abundance values for each category, therefore we assigned A = 30, C = 10, O = 3, and R = 1.

Abundance data, or numeric density, fails to represent overall fish assemblage composition, because all species are not equal in biomass. To overcome this limitation we chose a standard size estimate for each species to convert density to biomass; while assuming individual species of fish were uniform in size ignored obvious intra-specific variation, it emphasized clear size differences between species, such as large-bodied and small-bodied herbivores. The standard size estimate used was the size at first maturity (L_50_) based upon equations from FishBase and asymptotic lengths from ongoing fisheries-dependent datasets. L_50_ was chosen because it is a universal measure pertaining to life-history and emphasized inter-specific size differences.
